# Supplementary material for: Complexome profiling of the Chlamydomonas psb28 mutant reveals TEF5 as an early PSII assembly factor
Source: Plant Cell. 2025 Mar 17;37(6):koaf055. doi: 10.1093/plcell/koaf055 (PMC12164590; doi:10.1093/plcell/koaf055)
Supplement: koaf055_Supplementary_Data [file koaf055_supplementary_data.zip › psb28_interactive.zip!/plots/intensity/qualityPlots/peptides/_Cre09.g392616.t1.1.html]

### Cre09.g392616.t1.1

Trivial name:   
SeqCoverage [median]: 11.40  
Mass: 30.72 kDa  
  
 

| Sequence | MissedCleavage | Mass [Da] | wtIntensity | muIntensity | Score |
| --- | --- | --- | --- | --- | --- |
| AADLAAQAGK | 0 | 914.48 | 996.18 | 0.00 | 79.47 |
| EVGAGPQN | 0 | 770.36 | 1851.07 | 1270.49 | 138.48 |
|  |  |  | 2847.3 | 1270.5 |  |
